# A Treatise on the Diseases of Infancy and Childhood

**Published:** 1876-04

**Authors:** 


### Books Reviewed.

*A Treatise on the Diseases of Infancy and Childhood.* By J. LEWIS SMITH, M. D. Third Edition. Enlarged and thoroughly Revised. Philadelphia: Henry C. Lea. 1876. Buffalo: T. Butler & Son.

The urgent call for the third edition of Dr. Smith's excellent work is ample evidence that it is a favorite with the profession. In the present revised, enlarged and illustrated edition it will be found that several chapters have undergone more or less important modifications and changes. The chapter on diphtheria has, owing to the author's increased experience in that disease, been modified in several particulars. He does not agree with Oertel, and other German writers, in attaching great significance to the presence of micrococci in the false membrane of this affection.

Dr. Smith describes for the first time in an English text-book, we believe, what is known as Rothlen, or German measles. He also devotes a chapter to cerebro-spinal fever, which he considers as a general disease, the pathological changes of which are not, in a large number of cases, to be found in the brain or spinal cord. The text has been so arranged, and the size of the type so modified, that, notwithstanding a large amount of additional matter, the work is the same size as the former edition. It can safely be said that it is one of the best text-books in the English language on diseases of children.

